# Optimization of the Fluidic-Based Assembly for Three-Dimensional Construction of Multicellular Hydrogel Micro-Architecture in Mimicking Hepatic Lobule-like Tissues

**DOI:** 10.3390/mi12091129

**Published:** 2021-09-20

**Authors:** Qian Liang, Yaozhen Hou, Fei Meng, Huaping Wang

**Affiliations:** 1 Intelligent Robotics Institute, School of Mechatronical Engineering, Beijing Institute of Technology, Beijing 100081, China; liangqianbjpc@126.com (Q.L.); 3120195111@bit.edu.cn (Y.H.); 2 Beijing Advanced Innovation Center for Intelligent Robots and Systems, Beijing Institute of Technology, Beijing 100081, China; wanghuaping@bit.edu.cn

**Keywords:** tissue engineering, cell-laden microstructures, self-assembly, process optimization, co-culture

## Abstract

Three-dimensional (3D) assembly of microstructures encapsulating co-cultured multiple cells can highly recapitulate the in vivo tissues, which has a great prospect in tissue engineering and regenerative medicine. In order to fully mimic the in vivo architecture, the hydrogel microstructure needs to be designed into a special shape and spatially organized without damage, which is very challenging because of its limited mechanical properties. Here, we propose a 3D assembly method for the construction of liver lobule-like microstructures (a mimetic gear-like microstructure of liver lobule) through the local fluidic interaction. Although the method has been proven and is known as the consensual means for constructing 3D cellular models, it is still challenging to improve the assembly efficiency and the assembly success rate by adjusting the fluidic force of non-contact lifting and stacking. To improve the assembly efficiency and the assembly success rate, a fluidic simulation model is proposed based on the mechanism of the interaction between the microstructures and the fluid. By computing the simulation model, we found three main parameters that affect the assembly process; they are the velocity of the microflow, the tilt angle of the manipulator and the spacing between the microstructures and the manipulator. Compared with our previous work, the assembly efficiency was significantly improved 63.8% by using the optimized parameters of the model for assembly process, and the assembly success rate was improved from 98% to 99.5%. With the assistance of the assembly simulation, the luminal 3D micromodels of liver tissue show suitable bioactivity and biocompatibility after long-term hepatocytes culture. We anticipate that our method will be capable of improving the efficiency of the microstructures assembly to regenerate more complex multicellular constructs with unprecedented possibilities for future tissue engineering applications.

## 1. Introduction

Due to the rapid development of tissue engineering, three-dimensional cellular structures can be effectively fabricated with customized microstructural features and integrated morphology, which can recapitulate the cellular microenvironment in vivo and are widely applied in drug delivery, targeted therapy, organ transplantation and other fields [1,2,3,4,5,6]. The fabrication of artificial tissue can be divided into bottom-up and top-down assembly of three-dimensional microstructures. The top-down method involves the inoculation of a large number of proliferating cells in vitro onto a biomimetic microscaffold. With the segmentation, arrangement and recombination of the proliferative cells, the biomimetic three-dimensional microtissues were fabricated. However, there are some limits to the top-down method; for example, it is difficult to control the process of cell proliferation and extracellular matrix (ECM) secretion on microscaffold accurately. The permeation rate of proliferated cells into the microscaffold is low on account of the limitation of spatial resolution. The type of cells and the uniformity of proliferation cannot be precisely controlled on the microscaffold. In order to produce more complex tissues, the bottom-up assembly of repeated microstructures has emerged. The goal is to fabricate functional modules that mimic natural microstructures and assemble every independent module into a complete microtissue or an organ. The method can achieve more uniform cell distribution and more controllable cellular microenvironment in the engineered composites [7,8,9,10,11].

In past two decades, the spatial organization of microstructures by stacking, random packing or the field-driven force including magnetic field, optical field and electronic field have been widely applied [12,13,14]. Optical tweezers rely on a trap created by a highly focused laser beam to steer micron-sized particles or polymer spheres on a chip. Dielectrophoresis technology makes it possible to assemble irregular micro-building blocks based on optically induced electrodynamic chips. Magnetic assembly utilizes magnetic fields to control cellular microstructures embedded with magnetic nanoparticles [15,16,17,18,19,20]. All of the above assembly methods do not have enough assembly force to guarantee the stability and consistency of each assembly process, and also cannot arrange microstructures spatially and cannot achieve collective assembly for anisotropic microstructures, leading to low assembly efficiency and increased failure rate. Although the physical push can provide larger force to manipulate the microstructures and keep them regularly on the microneedle, the physical push cannot flexibly adjust the contact force and the biomaterial to fabricate the microstructures are very fragile, easily damaged when the micromanipulator contacts to them [21,22,23,24]. Thus, a more efficient method to implement the fluidic-based assembly process is proposed. This method can utilize the local fluidic force to flexibly grasp, arrange and stack microstructures in space. Because of the non-contact form of the assembly, it is very easy to manipulate the microstructures that are soft to avoid destruction. Nevertheless, the fluidic force still has a problem that it can be easily affected by external conditions and become unstable. A novel microfluidic device was fabricated with multiple Polydimethylsiloxane (PDMS) layers to construct vascular-like cellular structures. It utilized the microchannel in the device to guide the microstructures to achieve its continuous arrangement and stack. However, this method was limited in a closed environment, which makes the assembly method lack versatility [25]. To overcome the above difficulties, a self-assembly process that in an open environment utilizes the oriented fluidic force to assemble micro organizational units in a guided and scalable manner is desirable [26,27].

**Figure 1:**
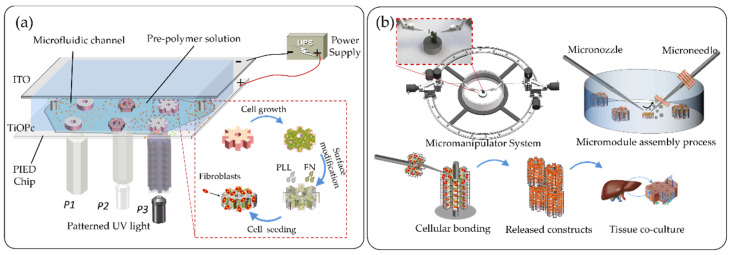
The schematic of microstructure biofabrication, assembly and co-culture. (**a**) Fabrication of lobule-like microstructures using photo-induced electrodeposition (PIED) system, with long-time cells co-culture and proliferation. (**b**) Assembly of three-dimensional (3D) lobule-like constructs using a coordinated micromanipulation system to achieve a hepatic functionality multicellular micromodel.

In this paper, we propose a fluidic-based assembly method to spatially integrate the fragile microstructures into a three-dimensional (3D) architecture through the integration guided with local fluidic force. As shown in Figure 1a, the microstructures are fabricated by electrodeposition of alginate hydrogel into a hollow structure embedding cells inside [19,27,28,29]. In Figure 1b, the cellular microstructures can be lightly lifted and stacked layer by layer into a lobule-like 3D micro-architecture along the micropillar through the coordination of dual micromanipulators. However, this assembly method, which is commonly used for constructing 3D cellular models, has obvious shortages [15,30,31,32,33]. As to the microstructures are mostly composed of hydrogels that have poor mechanical characteristics, the microstructures are easily damaged by the fluidic force of the assembly process. Moreover, the posture of the manipulators, the distance between the manipulators and objects and the dynamic parameters of the fluid will affect the assembly efficiency, the assembly success rate and the assembly automation. Therefore, it is meaningful to study the underlying mechanism of fluidic assembly process, find the key parameters that affect the assembly efficiency and the success rate and balance the relationship between them. We built a fluidic model to simulate the real assembly process and extract three important parameters that can be optimized for improving the assembly efficiency and the success rate, which are the velocity of the microflow, the tilt angle of the manipulator and the spacing between the microstructures and the manipulator. Compared with our previous work, we studied the underlying mechanism of the fluidic assembly process and extracted three main parameters that can be optimized to improve the efficiency and the success rate of the 3D cellular model assembly process. The results show that the assembly efficiency and the assembly success rate are significantly improved by using the optimized parameters. Moreover, the 3D cellular architecture with specific microstructural features and integrated morphology were fabricated and showed high cell viability and intercellular interactions in the long-term co-culture process. Overall, our optimization method provides accurate parameters for the process of assembling gear-like microstructures into biomimetic liver lobules and offers a significant impact and potential for three-dimensional microtissue construction.

## 2. Materials and Methods

### 2.1. Motion Analysis of Module

**Figure 2:**
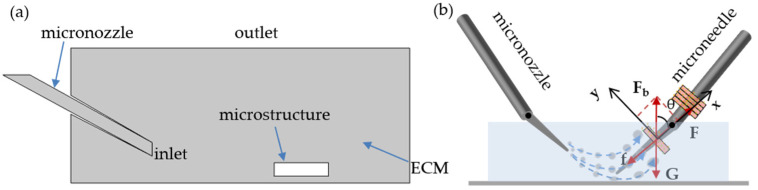
Schematic diagram of the assembly process. (**a**) The microflow blowing out from the inlet through the microstructure to the outlet. (**b**) Force analysis of the microstructure. During the assembly, the microstructure is subjected to gravity *G*, buoyancy *F*_b_, friction f and thrust *F*.

The motion analysis of microstructures plays a fundamental role in the subsequent modeling analysis and microstructure assembly. As shown in Figure 2b, the module on the microneedle remains stationary under gravity, buoyancy, viscous resistance and friction. In Equation (1):

(1) $$F=f+G(x)+F_{bx}$$

where F is the thrust of microflow, f is the static friction between the microneedle and microstructure, F*_b_* and G are the buoyancy and gravity of the microstructure itself, respectively, and seta is the angle between the microneedle and the plate. Force F is the key to the assembly process; it comes from pushing of the microflow. In this model, the speed of the microstructure in liquid is proportional to the sum of external forces at any time; therefore, an energy change in the microflow affects the efficiency of the assembly process. We analyzed three factors affecting the change in the microflow: the velocity of microflow, the angle and the distance between the two micromanipulators.

### 2.2. Simulation Model Creating

In order to optimize the method of assembly process, we chose COMSOL Multiphysics^®^ software (COMSOL Inc., Burlington, VT, USA) to make a model for simulating the assembly process and made several assumptions. Since the microneedle is fixed during the assembly process, the liver lobules can be approximated as moving in a vertical plane. We simplified the simulation model. In Figure 2a, a flat square was used to simulate the petri dish. A slender tube was considered the micropipette that blows out microflow. We used a small square to replace the lobule-like microstructure.

In this paper, we only simulate the pushing process by the microflow. Therefore, we hypothesized that the fluid is incompressible. By considering the microflow velocity and the microstructure speed, the Mach number, which is equal to the maximum speed of the fluid divided by the sound speed in the medium, is below 10^−6^ [26]. As we know, the Reynolds number is defined as Re=LVρ/γ, where *L* is the length of the object, *V* is the speed, *ρ* is the fluid density and γ is the fluid dynamic viscosity. In our model, the fluid inertia is negligible on account of the Re = 3 × 10^−2^, which is far below 1.

Since the module is suspended in the ECMs in the initial state, it is very important to adjust the parameters of microflow on the module in order to have the module threaded through the microneedle accurately and quickly [24,25]. Figure 2b indicates the force exerting on the module by microflow during the assembly process. Force *F* is generated by the microflow which was subjected to three types of parameters: the microflow’s velocity, the angle between the micropipette and the bottom plate and the distance between the micropipette and the microneedle. Adjusting strategies are as follows.

1. The velocity of microflow:
  The value of velocity determines the power of microflow and it affects the movement of the module directly. For analyzing velocity, parameters were 0.3 μm/s, 0.6 μm/s and 0.9 μm/s; we left the other two parameters (angles and distances) unchanged. The physical model is selected as the laminar flow interface.
2. The angle between the micronozzle and the bottom plate:
  The angle determines the trajectory of the microflow. The changes in trajectory could affect the force applied on the module. Therefore, it is essential to choose a proper tilt angle to obtain a trajectory for avoiding loss of the force. We only changed the tilt angle on 30, 45 and 85 degrees to observe the variations of trajectory.
3. The distance between the two micromanipulators:
  The microflow will lose energy due to the viscosity of fluid before pushing the module. In theory, the closer the micronozzle and the microneedle are, the greater force applied on the module, which is helpful to push the microstructure through the microneedle. We moved the micronozzle approach to the module at the distances of 18 mm, 16 mm and 14 mm and observed the force applied on the module.

## 3. Results and Discussions

### 3.1. Equipment Setup

The assembly simulation model was designed and optimized using the Level Set Two-Phase Flow Module provided by COMSOL Multiphysics 5.5. The photo-induced electrodeposition (PIED) system consists of three major components: a photoconductive electrodeposition chip, a visible light supply and a direct-current (DC) power supply, as shown in Figure 1a. In brief, the upper plate of the photoconductive electrodeposition chip is an Indium Tin Oxide (ITO) plate, connected to the cathode of the DC power; the lower plate is an ITO plate coated with titanyl phthalocyanine (TiO-Pc), and connected to the anode. There is a channel between two plates filled with alginate film, and a light source can be taken as an assist for electrodeposition to generate the film to obtain arbitrary shape microstructures. Light sources with various types of patterns can be modulated in real time by the Digital Micro-Mirror Device (DMD) system.

The coordinated micromanipulation system includes a set of dual micromanipulators and a circular rail, on which the stepper motors controlled the micromanipulators to move concentrically along the rail. A side of the dual micromanipulator was a glass rod (G-1, Narashige Inc., Tokyo, Japan) and the other side was a glass capillary (G-1000, Narashige Inc.). The glass capillary was used as the main effector and the untreated end was connected to a syringe pump for water injection. The glass rod was used as the sub-effector and was fixed opposite to the main effector. It was also used as a pole for microstructure assembly. Each micromanipulator was configured with three translational degrees of freedom (DOF) and is controlled by three piezoelectric motors (Model 8353, New Focus Inc, California, USA) with a resolution of approximately 30 nm. Thus, the tip of the micromanipulator can flexibly adjust its attitude within a limited microscope field of view during assembly.

### 3.2. Materials Preparation and Microstructure Fabrication

**Figure 3:**
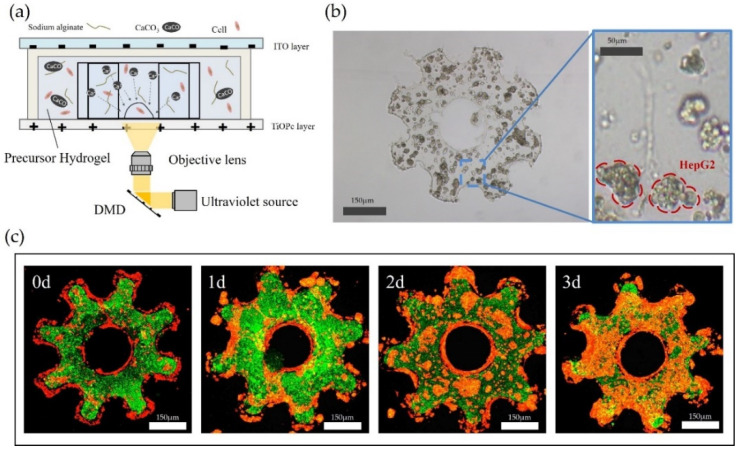
(**a**) Schematic of the microstructure fabrication based on PIED. (**b**) HepG2 cells culture in gear-like microstructure. (**c**) Fluorescence images of fibroblast (red) and hepatocyte (green) treated with Poly-L-Lysine (PLL) and fibronectin (FN). They are observed by scanning confocal microscopy for 0, 1, 2 and 3 days.

To produce shape-controlled alginate microstructures for constructing the mimicking lobule-like 3D constructs, we used the fabrication method based on electrodeposition, which is known as the basic consensual means of fabricating 3D cell structures. The Ca-alginate hydrogel electrodeposition process was programmable; the mechanism of PIED is illustrated in Figure 3a. After the electrodeposition, the remaining undeposited hydrogels on the ITO plate were washed off with deionized water, then the alginate hydrogel microstructures were collected. To mimic the natural multicellular environment, HepG2 cells and NIH/3T3 fibroblasts were used to modify the surface of the cell-laden microstructures, as shown in Figure 3b. The surface of the alginate-PLL-alginate (APA) layered microstructures was treated with Poly-L-Lysine (PLL, molecular weight: 30,000–70,000; Sigma-Aldrich, St. Louis, MO, USA) and fibronectin (FN) to enhance the adhesion of fibroblasts. In this process, PLL was easily bound to mannuronic acid and guluronic acid (M-G) blocks on the alginate surface to form bridges that connected with FN. Therefore, the cell-loaded APA microstructures were transformed into APA-PLL-FN microstructures, and the cell surface adhesion and proliferation were enhanced through the cell attachment area of FN. After cells seeding, fibroblasts proliferated and spread on the surface of the microstructures and gradually covered it, as shown in Figure 3c. For more detailed fabrication methods of microstructures, please refer to our previous research [19].

### 3.3. The Velocity of Microflow

**Figure 4:**
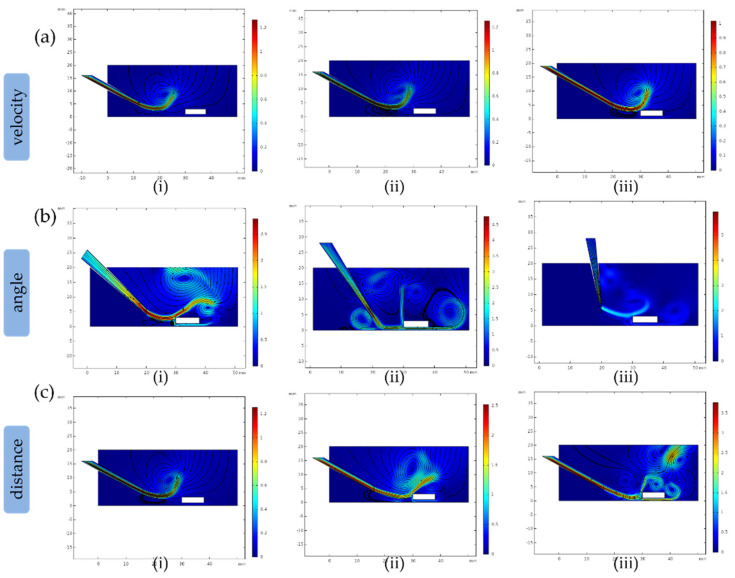
Simulation results of (**a**) microflow velocity; (**b**) microflow angle; (**c**) distance between the micromanipulators for assembly process.

**Figure 5:**
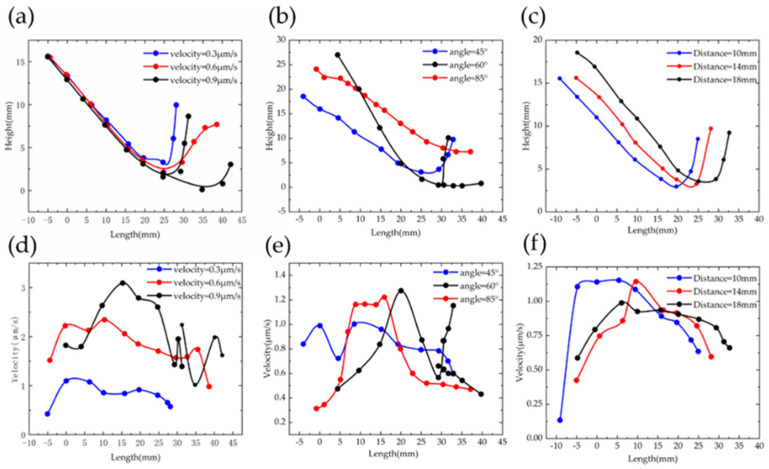
Trajectory data (**a**–**c**) and velocity rate (**d**–**f**) of the microflow relative to previous simulation.

In this article, the assembly process can be simulated by three sets of parameter combinations: the velocity of spraying microflow, the tilt angle of micronozzle and the space between two micromanipulators. Figure 4a shows the variation of the ejected microflow trajectory when the velocities were 0.3 μm/s, 0.6 μm/s and 0.9 μm/s. When the velocity of the microflow was 0.3 μm/s, Figure 4(ai) shows that the microflow trajectory did not reach the microstructure, and consequently, the microstructure was not lifted. Compared to the results of (ii) and (iii), both microflows reached the microstructure, and the streamline in (iii) produced a faster flow rate than (ii). As depicted in Figure 5a, the curves show that the longest spread distance is the microflow with the initial velocity of 0.9 μm/s. Figure 5d also indicates that the instantaneous velocity is 2.3 µm/s when x = 30 mm, which higher than the other two curves. We can conclude that higher the outlet velocity is, the more force the microstructure received. We finally chose the outlet velocity as 0.9 μm/s to guarantee that the microstructure was lifted by the sufficient force of the microflow.

### 3.4. The Tilt Angle of the Micropipette

In this regard, the tilt angle of the micronozzle determines the microflow trajectory and then changes the posture of the microstructure. It can be seen in Figure 4b that when the ejected microflow reached the microstructure, the microflow was divided into two parts: the upper one produced a horizontal thrust, and the lower part produced a lift force to raise up the microstructure. However, the microflow trajectories were not divided uniformly, as this depends on the tilt angle of the micronozzle. If less microflow went down to the microstructure, the microstructure would not be raised up by sufficient force. Furthermore, decreasing the tilt angle may have made the situation worse. To change this, we increased the tilt angle to 45° and 60°, and the efficacy of the microflow divided in Figure 4(bii) was better than the microflow trajectory in Figure 4(biii). Notably, because the clearances between the microstructure and the bottom were small, the tilt angle cannot be increased continually. As shown in Figure 4(biii), the tilt angle was 85°, and there was little microflow access at the bottom of the microstructure. The black plot in Figure 5b illustrates that the trajectory of the microflow with a tilt angle of 60 degrees can be divided and pushed the microstructure simultaneously. Note that for the velocity of the microflow with 60 degrees in (e), the instantaneous velocity of microflow on the surface of the microstructure (x = 30 mm) was 0.68 μm/s and 0.83 μm/s, better than the other two groups of data. In short, if the directions of the resultant force of the two microflows were exactly upwards along the microneedle, and the value of the force was sufficient to push the structure, a better assembly efficacy can be achieved.

### 3.5. The Spacing between Two Manipulators

It is easily conceivable that the horizontal space between the two manipulators determines the energy consumption of the microflow. Generally speaking, decreasing the spacing is helpful to retain more kinetic energy for assembly. Furthermore, at the micro scale, the parameters changing may cause a significant disturbance to the assembly process. Based on the previous results we have discussed, changing the set parameters would influence the instability of the microflow and increase the debugging of the model. Therefore, we maintained the other two parameters (velocity = 0.9 μm/s, angle = 60°). When the distance was set to 14 mm and 16 mm, the trajectories of the microflow in Figure 4(ci,cii) did not reach the microstructure (x = 30 mm). From the Figure 5c, the plot tended to be closer to the microstructure with the set distance increasing. When we turned the distance to 18 mm, the front end of the microflow just reached the microstructure, as shown in Figure 4(ciii). Remarkably, the data of the microflow in Figure 5f also verified our hypothesis before. The microstructure (x = 30 mm) could acquire more energy exerted by the microflow (black line), and the velocity was 0.88 μm/s.

### 3.6. Module Assembly

**Figure 6:**
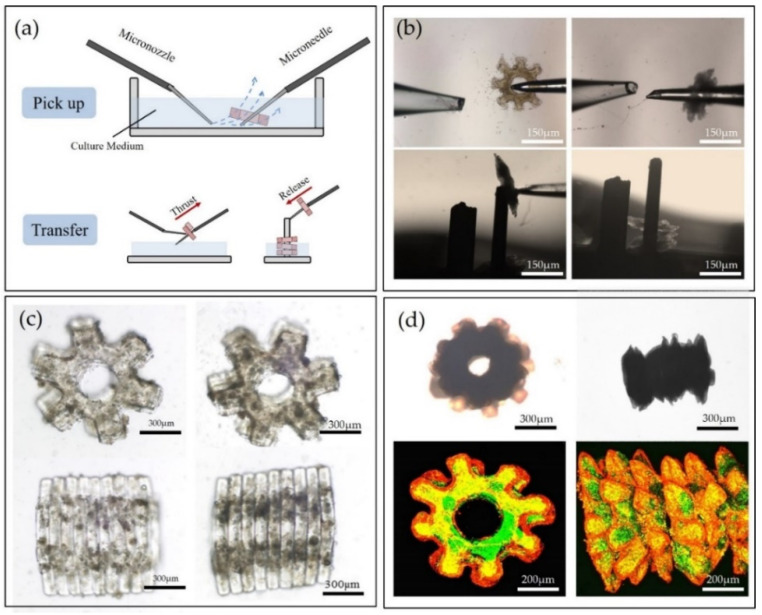
(**a**) Schematic of microstructure assembly. It concludes two steps of pick-up and transfer. (**b**) Real-time assembly process with pick-up and transfer. (**c**) Top and side views of released three-dimensional co-culture constructs. (**d**) Optical and fluorescent images of three-dimensional lobule-like constructs coated with hepatocyte and fibroblast.

To allow hepatocytes to survive in the natural hepatic lobules, the mimic lobule three-dimensional microstructures with built-in vessels need to be assembled. The working principle of picking-up and transferring microstructures by the micromanipulators are shown in Figure 6a. The assembly method in our previous research [19] was mainly based on the visual feedback for remotely assembling the microstructures to construct a 3D tissue-like model. Since the previous method lacked the precise control of the assembly parameters such as fluidic force, manipulation angle and distance, the assembly efficiency and assembly success rate have potential to improve. In order to achieve the assembly automation, the underlying mechanism of the fluidic-based assembly process should be cleared, and further extracting the key parameters that can be optimized for improving the assembly efficiency and the success rate. Thus, we built a fluidic model to simulate the real 3D cellular assembly process and concluded three assembly parameters of the velocity of microflow, the intersection angle of the manipulator and the microstructures and the distance between the two manipulators. In the pick-up process, we put microstructures onto the rod easily by choosing appropriate combinations of the assembly parameters on the basis of the simulation results. In the following transfer process, the microstructure was transferred into the micropillar. Repeating the process, microstructures could be assembled into three-dimensional constructs continuously by gravity. Compared with our previous results, the efficiency of the assembly was improved by 63.8%, and the success rate of the assembly are improved from 98% to 99.5% (see Figures S1–S5 in the Supplementary Materials). Figure 6b illustrated the pick-up and transfer process in assembly.

After assembly, the microstructures coated with proliferated fibroblasts (green fluorescent area) and then secreted ECM as biological glue to bond the adjacent microstructures together. As shown in Figure 6c, there are no gaps between adjacent microstructures and the microstructures can be arranged tight and regular under the guidance of the micropillar. As depicted in Figure 6d, we spread HepG2 cells on the surface of the three-dimensional constructs. After 3 days culture, the hepatocytes cells gradually differentiated and wrapped around the constructs. The red areas in this fluorescent image showed the proliferation of HepG2 cells clearly. It is believed that, compared with the conventional assembly method, our assembly method fabricated more aligned and regular three-dimensional liver lobule-like tissues with less deformation in an efficient manner (see the methods for measuring cell viability in the supplementary). In order to evaluate the assembled 3D constructs that possess the partial function of the real liver lobule, we previously evaluated the albumin secretion and urea synthesis. We also previously established perfusion experiments by perfusing APAP (acetaminophen) through the built-in lumen of the 3D lobule-like construct, which can mimic the drug distribution and diffusion in native liver as much as possible (see the Figure S6–S8 in Supplementary Materials). The results indicated that the 3D model with tissue-specific morphology can provide a suitable environment to improve cell growth and biofunction in vitro (see Refs. [15,19,29]). Although the cultured 3D construct cannot demonstrate enough biological features of the real liver tissues, it shows part of the behavior, such as the albumin secretion and urea synthesis. The current constraints come from the differences between our building blocks (the units for assembly) and the in vivo units of tissue; we will further collaborate with researchers in biology field who can provide us more biomimetic units fabricated by a professional method. Thus, we can assemble the more realistic microstructure units to achieve a bottom-up 3D integration by our assembly method, and even produce the biological substitute of real tissue.

## 4. Conclusions

We successfully developed a simulation model of the assembly process. Through adjusting the parameters, we found that the velocity of the microfluid is the key parameter affecting the assembly process. We increased the velocity of the microflow to exert more thrust, which is helpful to thread the microstructure onto the microneedle quickly. Then, we changed the tilt angle of the micronozzle to adjust the trajectory of the microflow. We found that a proper tilt angle can thread the microstructure onto the microneedle accurately. At last, we found an appropriate working space of the microflow by adjusting the distance between two micromanipulators. The results showed that the closer two micromanipulators were, the easier the microstructures were threaded. Compare with our previous work [19], the efficiency and the success rate of the assembly process were significantly improved through the analysis of the underlying mechanism of the interaction between the fluidics and the microstructures and the optimization of the related key parameters. After the long-term culture of the fibroblasts and hepatocytes, and the cell ability assessment of the luminal three-dimensional constructs, they showed good bioactivity and biocompatibility. For future works, we will build simulation models of a variety of three-dimensional microtissues assembly to enhance the generality and accuracy of the model prediction. Moreover, the diversity of the physical and chemical properties of the microstructures and the surrounding microenvironment may influence the assembly process, which should be considered. We will focus on enriching the biological functionality of 3D constructs, improving the cell viability and improving the efficiency of intercellular interactions.

## Data Availability

The processed data required to reproduce these findings cannot be shared at this time as the data also forms part of an ongoing study.

## Supplementary Materials

The following are available online at https://www.mdpi.com/article/10.3390/mi12091129/s1, Figure S1: Cell viability of fibroblasts and hepatocytes after the assembly process. In the experiment, all 3D constructs were cultured at 37 °C under 5% CO2 and were treated with 0.05% PLL. After 4 days co-culture, the cell viability above 92%. The values represent the mean ± SD from five independent experiments. Figure S2: Cells encapsulated in the microstructure were stained with calcein AM (green, live cells) and propidium iodide (red, dead cells) after co-culturing 7 day. Figure S3: Fluorescent images of live (green)/dead (red) assay of 3D lobule-like constructs after culturing 7 days. Figure S4: The success rate of the assembly on different shapes of microstructures Figure S5: The assembly time on different shapes of microstructures Figure S6: The assembly results of multiple shapes of microstructures. Figure S7: The assembly time comparison of previous and the present method. The values represent the mean ± SD from five independent experiments. Figure S8: The assembly success rate comparison of previous and the present method. The values represent the mean ± SD from five independent experiments.
