# Supplementary material for: Optimization of the Fluidic-Based Assembly for Three-Dimensional Construction of Multicellular Hydrogel Micro-Architecture in Mimicking Hepatic Lobule-like Tissues
Source: Micromachines (Basel). 2021 Sep 20;12(9):1129. doi: 10.3390/mi12091129 (PMC8471618; doi:10.3390/mi12091129)
Supplement: Supplementary file 1 [file micromachines-12-01129-s001.zip › micromachines-1300949-supplementary.pdf]

micromachines

Supplementary Materials: Optimization of the fluidic-based assembly for three-dimensional construction of multicellular hydrogel micro-architecture in mimicking hepatic lobule-like tissues

Qian Liang [1,†], Yaozhen Hou [1,†], Fei Meng [1,*] and Huaping Wang [2]

1 Intelligent Robotics Institute, School of Mechatronical Engineering, Beijing Institute of Technology, Beijing 100081, China; liangqianbjpc@126.com (Q.L.); 3120195111@bit.edu.cn (Y.H.)
2 Beijing Advanced Innovation Center for Intelligent Robots and Systems, Beijing Institute of Technology, Beijing 100081, China; wanghuaping@bit.edu.cn
* Correspondence: mfly0208@bit.edu.cn; Tel.: +86-010-68917626
† These authors contributed equally to this work and are both the first author.

1. Methods

Cell Viability Assay:

Live/dead staining assays of the hydrogel micromodules were performed by labeling with calcein AM (2 μg/mL) and propidium iodide (3 μg/mL) fluorescent dyes (Sigma, St. Louis, MO, USA). The cell viability in different micromodules was evaluated daily, and the areas of red fluorescence (dead cells) and green fluorescence (live cells) in each image were analyzed by ImageJ software. The cell viability in each group was represented by the green fluorescence area relative to the whole fluorescence area.

Cell Counting:

Before cell counting, the micromodules were washed twice with the HEPES buffer solution to remove the culture medium. The cell numbers in the micromodules were directly counted using a Cell Counting Kit-8 (Sigma). For co-cultures, the cell numbers in the micromodules were counted with the Cell Counting Kit-8 after enzymatic digestion with trypsin-EDTA (Gibco) for 5 min to remove the coated fibroblasts.

2. Evaluation of the Assembly Process

All data were represented as means ± standard deviation (SD).

Viable analysis of the assembly process:

Since the picked microstructure is suspended on the microneedle, the amount of the units in one stack theoretically depends on the length of the microneedle immersed in the liquid. In the actual assembly process, the assembled 3D constructs with the height from a few hundred microns to a few millimetres. To construct the 3D cellular model mimicking the microtissue, we always assembled about 10 units on a stack. However, as we mentioned, the amount of the units can be flexibly adjusted based on the requirement. In order to enable the assembled 3D constructs to mimic the morphology of real microtissue, we need to assemble several stacks and integrate them to achieve scale-up. Therefore, we assembled 8 to 10 repeating stacks in each assembly experiment, even we have assembled 15 stacks to achieve a 3D scale-up cellular model. Similarly, the amount of the stacks can be beforehand determined according to the size of the 3D cellular model, which can be flexibly adjusted during the assembly process. In our assembly process, the microneedle automatically approaches to the target and locates at the central of the microstructure and make the microneedle apply a gentle force down to the microstructure to make it upwarp. And then the micronozzle approaches to the microstructure from another side to blow out the microflow. Consequently, the microstructure will be transferred to the microneedle.

Therefore, the main factors that influences the percentage of units that is not viable is the damage caused by three reasons: the system positioning deviation caused by the individual variations of the microstructures, the structural damage of the microstructures caused by the physical contact of the microneedle, and the poor mechanical strength of the microstructures. Actually, we previously have quantified the success rate of the assembled stacks, we took 20 groups of automatic assembly experiments on multiple shapes of microstructures, such as round, gear, triangle, and hexagon, where the number of microstructures in each assembly was 10. The final statistics showed that the success rate of the assembly was above 96%.

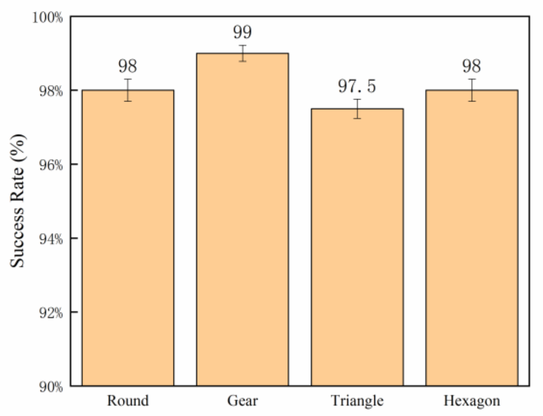

Figure S1. The success rate of the assembly on different shapes of microstructures.

A stack can be achieved by assembling several repeating microstructure units. In our assembly process, the microneedle automatically approaches to the target and locates at the central of the microstructure and make the microneedle apply a gentle force down to the microstructure to make it upwarp. And then the micronozzle approaches to the microstructure from another side to blow out the microflow. Consequently, the microstructure will be transferred to the microneedle. For evaluating the duration of assembling one stack, we decided to assemble 10 units with the shapes of round, gear, triangle, and hexagon. The assembly of each shape was repeated 5 times, where the mean duration of assembling gear-like microstructures to a stack was around 38 seconds.

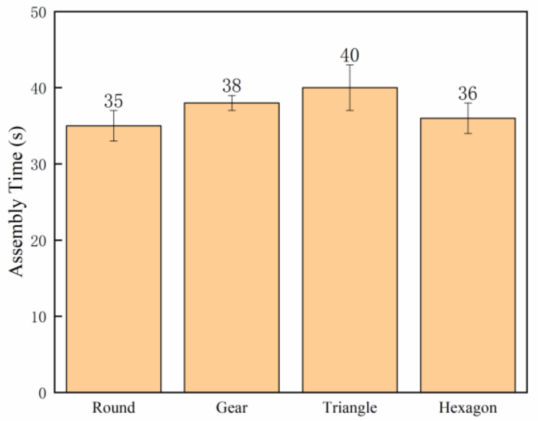

Figure S2. The assembly time on different shapes of microstructures.

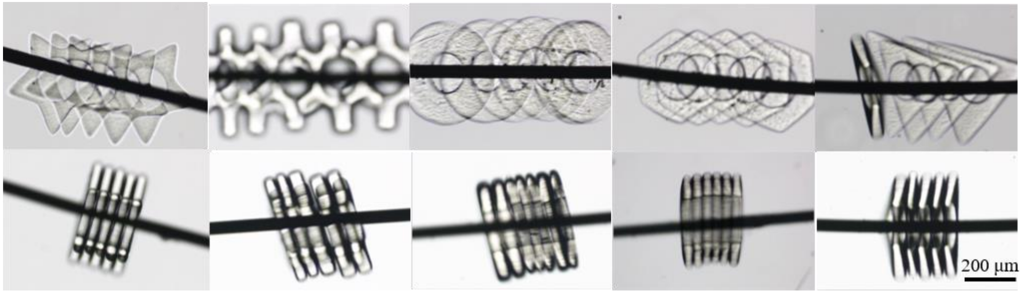

Figure S3. The assembly results of multiple shapes of microstructures.

Compared with our previous research [18], the assembly method in this paper has been simulated by a fluidic model, we found out and optimized the key assembly parameters that affecting the efficiency and the success rate of the assembly process, including the velocity of microflow, the tilt angle of the manipulator and the spacing between two manipulators respectively. By comparison, the results showed the significant improvement on the assembly success efficiency and the success rate.

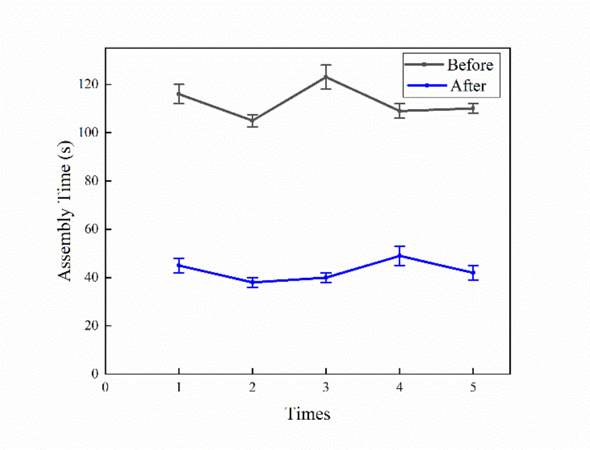

Figure S4. The assembly time comparison of previous and the present method. The values represent the mean ± SD from five independent experiments.

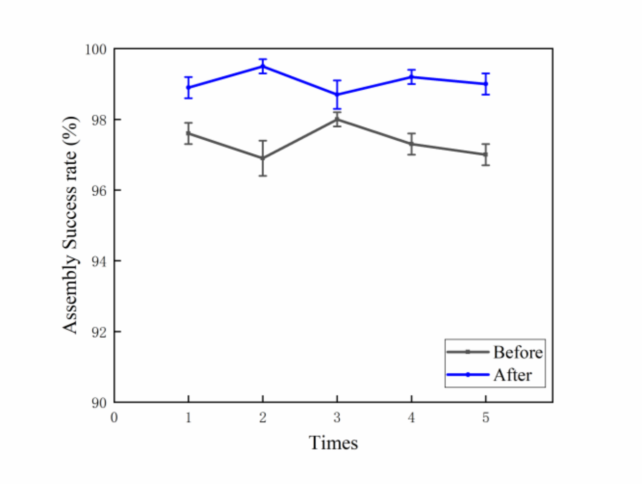

Figure S5. The assembly success rate comparison of previous and the present method. The values represent the mean ± SD from five independent experiments.

Functionality of the assembled 3D constructs:

We encapsulated the hepatocytes in a gear-like microstructure to mimic the modular unit of the hepatic lobule. Through the automated assembly manipulation, the repeating

microstructures were stacked into a radial 3D construct with lobule-like morphology and built-in lumen. The 3D constructs provide a lobule-like radial environment for cell grows and cell-cell interactions. The building lumen mimics the central vein and allows the nutrition perfusion. After few days co-culture, the cells could proliferate and secrete ECM as a bioglue to make the adjacent microstructures bond together. In order to evaluate the assembled 3D constructs that possess the partial function of the real liver lobule, we have evaluated the albumin secretion and urea synthesis before. Moreover, we also established perfusion experiments before by perfusing APAP (acetaminophen) through the built-in lumen of the 3D lobule-like construct, which can mimic the drug distribution and diffusion in native liver as much as possible. The results indicated that the 3D model with tissue-specific morphology can provide a suitable environment to improve cell growth and biofunction in vitro. Both of the experiments were published in our previous work. (see ref. [15,19,29] in manuscript).

3. Cell Viability Tests

In order to verify our fluidic-based assembly method is friendly to the microstructures and three-dimensional architecture, and will not produce side effects for cells co-culture or artificial tissue construction, we evaluated the co-cultured cells viability of fibroblasts and hepatocytes with 5 batches of independent experiments, each batch contained 20 groups. All the samples were incubated at 37 °C with 5% CO_2, and the culture medium was changed daily. Data were plotted as the mean ± standard deviation (SD) using Origin software.

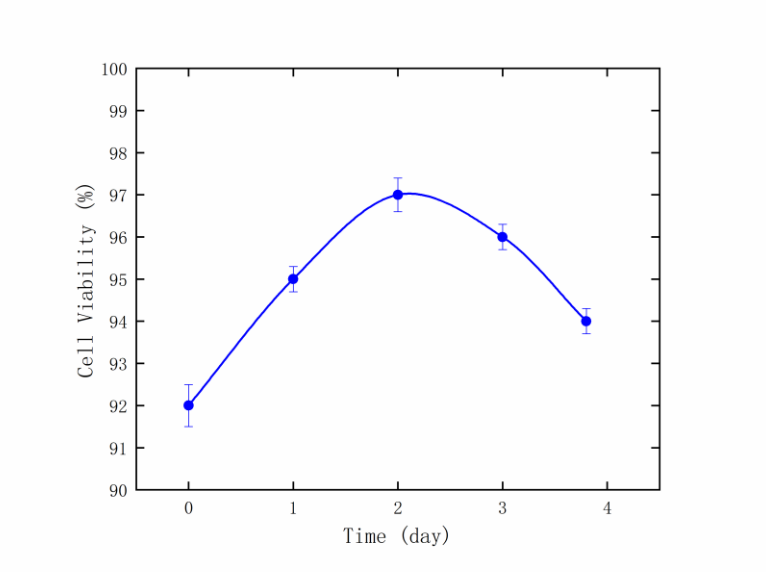

Figure S6. Cell viability of fibroblasts and hepatocytes after the assembly process. In the experiment, all 3D constructs were cultured at 37 °C under 5% CO_2 and were treated with 0.05% PLL. After 4 days co-culture, the cell viability above 92%. The values represent the mean ± SD from five independent experiments.

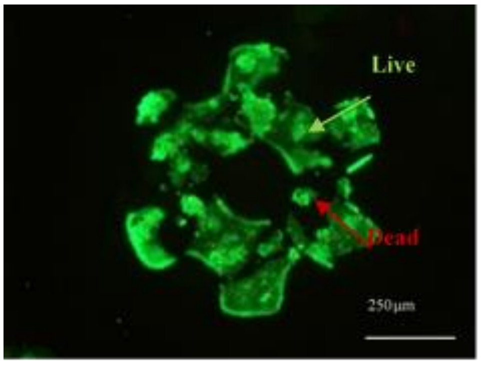

Figure S7. Cells encapsulated in the microstructure were stained with calcein AM (green, live cells) and propidium iodide (red, dead cells) after co-culturing 7 day.

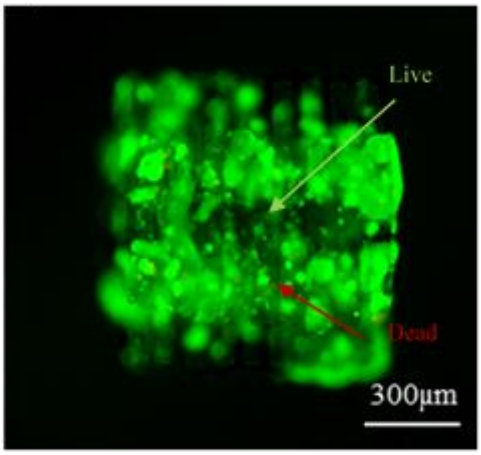

Figure S8. Fluorescent images of live (green)/dead (red) assay of 3D lobule-like constructs after culturing 7 days.
